# A Sociodemographic Profile of Mask Use During the COVID-19 Outbreak Among Young and Elderly Individuals in Brazil: Online Survey Study

**DOI:** 10.2196/28989

**Published:** 2021-09-14

**Authors:** Rodrigo L Vancini, Luiz Camargo-Neto, Marilia S Andrade, Claudio A de Lira, Rafaela G dos Santos, Pantelis T Nikolaidis, Beat Knechtle, Luiz HV Piacezzi, Maria CB Teixeira-Lopes, Ruth E Assayag-Batista, Meiry F Pinto-Okuno, Cássia R Vancini-Campanharo

**Affiliations:** 1 Universidade Federal do Espírito Santo Espírito Santo Brazil; 2 Escola Paulista de Enfermagem Universidade Federal de São Paulo São Paulo Brazil; 3 Departamento de Fisiologia Universidade Federal de São Paulo São Paulo Brazil; 4 Setor de Fisiologia Humana e do Exercício Faculdade de Educação Física e Dança Universidade Federal de Goiás Goiânia Brazil; 5 Campus X, Teixeira de Freitas Universidade do Estado da Bahia Bahia Brazil; 6 School of Health and Caring Sciences University of West Attica Athens Greece; 7 Institute of Primary Care University of Zurich Zürich Switzerland; 8 Medbase St. Gallen Am Vadianplat St. Gallen Switzerland

**Keywords:** aging, older adults, COVID-19, pandemic, sociodemographic profile, face mask, social media, online media, adolescents

## Abstract

**Background:**

Sociodemographic variables may impact decision making regarding safety measures. The use and selection of adequate face masks is a safety and health measure that could help minimize the spread of COVID-19 infection.

**Objective:**

This study aims to examine sociodemographic variables and factors relating to COVID-19 that could impact decision making or the choice to use or not use face masks in the prevention and care of a possible COVID-19 infection among a large sample of younger and older Brazilian people.

**Methods:**

An online survey composed of 14 closed-ended questions about sociodemographic variables and COVID-19 was used. A total of 2673 participants consisted of Brazilian people (aged ≥18 years) from different states of Brazil and were grouped according to age (≤59 years and ≥60 years). To compare the variables of interest (associated with wearing a face mask or not), chi-square and likelihood ratio tests were used (with P<.05 being significant).

**Results:**

Most of the participants in both groups were women from the southeast region who had postgraduate degrees. Approximately 61% (1452/2378) of individuals aged ≤59 years and 67.8% (200/295) of those aged ≥60 years were not health professionals. In the group aged ≤59 years, 83.4% (1983/2378) did not show COVID-19 signs and symptoms, and 97.3% (2314/2378) were not diagnosed with COVID-19. In the older adult group, 92.5% (273/295) did not show signs and symptoms of COVID-19, and 98.3% (290/295) were not diagnosed with the disease. The majority of the participants in both groups reported using face masks, and their decision to use face masks was influenced by the level of education and their occupation as a health professional.

**Conclusions:**

Younger and older adults have worn face masks during the COVID-19 outbreak. It is difficult to measure how much of a positive impact this attitude, habit, and behavior could have on the degree of infection and spread of the disease. However, it can be a positive indicator of adherence to the population’s security and safety measures during the pandemic.

## Introduction

COVID-19 is an infectious disease caused by SARS-CoV-2, which mainly affects the functioning of the pulmonary system [1]. Current evidence shows that the COVID-19 pandemic has a much higher mortality rate in older adults due to morbidities and bad lifestyle (poor diet and physical inactivity) associated with aging [2]. Factors associated with increased risk of mortality in COVID-19 include comorbidities related to aging, such as obesity, insulin resistance, and cardiovascular disorders, and individuals, especially older adults, are at risk of having lower functional capacity and physical activity levels [3-5], which makes them more vulnerable to the infection [2,4].

During periods of social isolation and physical distancing for a pandemic, coping strategies (physical activity and adequate diet) and safety measures (use of face masks) that promote well-being and improve or maintain the general state of health should be performed and encouraged among the general population to mitigate the negative impacts of the disease [6].

The COVID-19 pandemic has had an unprecedented impact on the global health, economy, and functioning of societies [7]. Latin American countries with a high level of social and economic inequality have experienced worse effects of the pandemic. For example, São Paulo, Brazil has a high population density and great disparity in demographic and epidemiological profiles, social and economic levels, and accessibility to health services and protection and safety measures, such as the use of face masks [8].

Therefore, understanding the sociodemographic variables and adherence to mask use is essential to auxiliary health decisions by public and health authorities to minimize the effects and negative consequences of the COVID-19 pandemic on public health [9]. The assessment of sociodemographic variables determines the impact of population diversity on the effects of the pandemic [10] and how choices regarding safety, security measures, and adherence could affect the consequences and outcomes in the field of public health.

It is known that a population’s profile and social characteristics can impact safety measures, hygiene, social distance, outcomes, and possible changes in timing of pandemics [11]. Due to the risk and characteristics of the professional practice and health literacy, it is quite plausible that health professionals, because of their frequent contact with the public, are more sensitive regarding adherence to safety measures [12]. Moreover, the physical and social distancing, use of face masks, and eye protection devices in public and health care facilities are needed for the public and health professionals to control COVID-19 transmission [13].

However, in low-income countries like Brazil, the lack of literacy and access to health care, the spread of fake news among the general population, and the lack of proper management by the government can make a difference in the results and outcomes of such complex health scenarios [14]. Unfortunately, this is what we observed in Brazil during the COVID-19 pandemic. Brazil has been one of the countries with the worst management of the public health crisis; it is on the brink of a progressive social and economic collapse. Thus, studies that aim to check the population’s sociodemographic profile and the choices related to security and protective measures during a pandemic are essential to make more precise decisions based on technical and scientific knowledge.

An online-based cross-sectional study involving 12 Bangladeshi residents 64 years of age recruited via social media investigated the influence of factors related to perception, knowledge, attitudes, and practices regarding COVID-19 and found that the factors that impact perception and choices were female sex, older age, higher education, higher family income, and urban area residence [15]. A cross-sectional survey conducted by telephone or mobile phone with older individuals living in the state of Minas Gerais, Brazil showed that older individuals were knowledgeable and had good health literacy regarding COVID-19, but those that did not implement all the preventive measures were older adult male individuals living by themselves with a low educational level, and they are more vulnerable to COVID-19 [16].

Importantly, individuals 60 years and older are more vulnerable to COVID-19, and the use of face masks is a protective, safety, and health care measure to decrease the risk and spread of infection [13]. In addition, older people have a higher peak viral load and, especially those with comorbidities, have higher mortality rates related to COVID-19 than young people. This would be associated with chronic inflammation present in older people who are frail, which could allow a “more effective” action from SARS-CoV-2 leading to serious infection-related complications [17].

Currently, epidemiologists emphasize that the use of face masks covering the mouth and nose effectively stops airborne infections. In general, health and government officials followed the World Health Organization recommendations and, in some cases, forced the population to wear face masks in public places. However, in some countries like Brazil, there is a low rate of health literacy among government officials at all levels. Research shows that using the right type of face mask, according to location and profession, protects and reduces the infection risk [18]. Our aim is to present the sociodemographic and economic profile and health features (about COVID-19) and compare the individual determinants of face mask use during the COVID-19 outbreak among younger and older Brazilian people.

## Methods

### Study Design

This online and cross-sectional survey was conducted between May 1, 2020, and May 31, 2020, and was approved by the Research Ethics Committee of the Federal University of São Paulo (Certificate of Presentation of Ethical Appreciation - CAAE: 31540620.9.0000.5505). The study was conducted according to the guidelines of the Declaration of Helsinki. We enrolled Brazilian citizens living in Brazil who were 18 years or older (to compose the groups ≤59 years vs ≥60 years), and the consent to participate was obtained from all participants. Participants who did not answer all the research questions were excluded to minimize discrepancies in the number of answers. However, the exclusion of unanswered questionnaires was low, as all the questions on the Google Forms were mandatory. Furthermore, minors were excluded because the aims and procedures were designed for adults.

### Participants

The sample was selected using a nonrandom method and comprised of 2673 participants (mean age 40.0, SD 13.8 years). These participants were divided into two age groups: ≤59 years (n=2378, 89%) and ≥60 years (n=295, 11%).

### Procedures

Participants were invited to answer the questionnaire voluntarily through posts made on social media (Facebook and Instagram) and WhatsApp using a standard text that publicized the study and drew attention to the importance of understanding the behavior of the population in relation to COVID-19, which could provide subsidies to implement awareness actions, reducing the spread of the disease.

A link to the informed consent form was provided to each participant. Upon consent and agreement to voluntary participation, the participants were directed to the mandatory study questions. The questionnaire was developed by the researchers; corrected and adjusted by a panel of health experts, including a professional statistician; made available on the online platform Google Forms; and disseminated through social networks. The online questionnaire [19] was composed of 14 closed-ended questions about the following variables: age, sex, origin, marital status, religion, family income, education, presence of signs or symptoms or confirmed diagnosis of COVID-19, and occupation as a health professional (yes or no). In addition, multiple-choice questions about COVID-19 included the following: knowledge about the forms of transmission of COVID-19; risk groups, signs and symptoms, and what to do if they were present; preventive measures (hand hygiene, use of masks, and cleaning of surfaces) to be taken in case of traveling; and information on popular beliefs regarding the prevention, transmission, and treatment of COVID-19.

### Statistical Analysis

For the descriptive analysis of categorical variables, frequency and percentage were calculated. For continuous variables, mean, SD, median, minimum, and maximum were calculated. To compare the variables of interest (associated with mask use), chi-square and likelihood ratio test (only for comparisons regarding the level of education) were used. A significance level of 5% (*P* value<.05) was used. The data were analyzed using SPSS, version 19 (IBM Corp).

## Results

Our sample consisted of the adult Brazilian population from different states of Brazil: Acre (n=1), Alagoas (n=3), Amapá (n=3), Amazonas (n=1), Bahia (n=23), Ceará (n=36), Distrito Federal (n=22), Espírito Santo (n=32), Goiás (n=31), Maranhão (n=23), Mato Grosso (n=31), Mato Grosso do Sul (n=13), Minas Gerais (n=109), Pará (n=13), Paraíba (n=12), Paraná (n=52), Pernambuco (n=2), Piauí (n=2), Rio de Janeiro (n=73), Rio Grande do Norte (n=8), Rio Grande do Sul (n=24), Rondônia (n=1), Roraima (n=5), Santa Catarina (n=30), São Paulo (n=2117; in the state of São Paulo, the use of facial masks became mandatory on May 7, 2020), Sergipe (n=2), and Tocantins (n=4). The majority of participants were from São Paulo.

Table 1 shows that most of the 2673 individuals in the study were aged ≤59 years (n=2378, 88.9%), were female (n=2039, 76.3%), were from the city of São Paulo (n=2331, 87.2%), were married (n=1261, 47.2%), were Catholic (n=1241, 46.4%), had a monthly family income of 4 to 7 minimum wages in R$ (n=813, 30.4%), and had completed or were currently doing postgraduate studies (n=1181, 44.2%). In addition, 61.8% (n=1652) were not health workers, 84.4% (n=2256) did not show symptoms of COVID-19, and 97.4% (n=2604) had no confirmed diagnosis of the disease. The studied age groups (≤59 years and ≥60 years) were not homogeneous regarding the following variables: sex, Brazil region, marital status, religion, total family income, professional type, and health characteristics and status (Table 1).

Table 2 compares the profiles of participants with regard to the use of masks between the groups in terms of sex (male vs female), education level, occupation as a health worker (yes vs no), and presence of signs and symptoms or a confirmed diagnosis of COVID-19 (yes vs no).

On the use of masks, there were significant differences in sex (female vs male; *P*<.001), level of education (*P*<.001), and occupation as a health professional (*P*=.001). Females, health professionals, and those with a higher level of education adhered to the use of masks more than others.

**Table 1:** Characteristics of sociodemographic variables of younger and older people during the COVID-19 outbreak.

Variables		Age group (years)		Total (N=2673), n (%)
		≤59 (n=2378), n (%)	≥60 (n=295), n (%)	
**Sex**				
	Women	*1833 (77.1)^a^*	*206 (69.8)*	2039 (76.3)
	Men	545 (22.9)	89 (30.2)	634 (23.7)
**Brazil region**				
	North	22 (0.9)	6 (2)	28 (1)
	Northeast	108 (4.5)	3 (1)	111 (4.2)
	Midwest	93 (3.9)	4 (1.4)	97 (3.6)
	Southeast (São Paulo is located)	*2060 (86.6)*	*271 (91.9)*	2331 (87.2)
	South	95 (4)	11 (3.7)	106 (4)
**Marital status**				
	Married	*1093 (46)*	*168 (56.9)*	1261 (47.2)
	Divorced	153 (6.4)	49 (16.6)	202 (7.6)
	Single	894 (37.6)	23 (7.8)	917 (34.3)
	Stable union	225 (9.5)	24 (8.1)	249 (9.3)
	Widowed	13 (0.5)	31 (10.5)	44 (1.6)
**Religion**				
	Catholic	*1080 (45.4)*	*161 (54.6)*	1241 (46.4)
	Evangelical	401 (16.9)	17 (5.8)	418 (15.6)
	Spiritism	386 (16.2)	60 (20.3)	446 (16.7)
	Agnostic and atheist	274 (11.5)	21 (7.1)	295 (11)
	Others	237 (10)	36 (12.2)	273 (10.2)
**Total family income in minimum wages (R$ monthly)**				
	<1	99 (4.2)	1 (0.3)	100 (3.7)
	1-3	620 (26.1)	56 (19)	676 (25.3)
	4-7	*730 (30.7)*	83 (28.1)	813 (30.4)
	8-10	339 (14.3)	50 (16.9)	389 (14.6)
	>10	590 (24.8)	*105 (35.6)*	695 (26)
**Education level according to the Brazilian standard**				
	Incomplete elementary school	5 (0.2)	1 (0.3)	6 (0.2)
	Complete primary education	15 (0.6)	2 (0.7)	17 (0.6)
	Incomplete high school	26 (1.1)	5 (1.7)	31 (1.2)
	Complete high school	245 (10.3)	47 (15.9)	292 (10.9)
	Higher education (or studying)	1025 (43.1)	*121 (41)*	1146 (42.9)
	Postgraduate studies	*1062 (44.7)*	119 (40.3)	1181 (44.2)
**Are you a health professional?**				
	No	*1452 (61.1)*	*200 (67.8)*	1652 (61.8)
	Yes	926 (38.9)	95 (32.2)	1021 (38.2)
**Have you experienced symptoms of COVID-19?**				
	No	*1983 (83.4)*	*273 (92.5)*	2256 (84.4)
	Yes	395 (16.6)	22 (7.5)	417 (15.6)
**Did you have a confirmed diagnosis for COVID-19?**				
	No	*2314 (97.3)*	*290 (98.3)*	2604 (97.4)
	Yes	64 (2.7)	5 (1.7)	69 (2.6)

^a^Data in italics were the most frequent.

**Table 2:** Characteristics of mask use according to the variables of interest during the COVID-19 outbreak.

Variables		Mask use, n (%)		Total, n (%)	*P* value
		No	Yes		
**Age (years)**					.80
	≤59	121 (5.1)	*2257 (94.9)^a^*	2378 (100)	
	≥60	14 (4.7)	*281 (95.3)*	295 (100)	
	Total	135 (5.1)	2538 (94.9)	2673 (100)	
**Gender**					<.001^b^
	Female	87 (4.3)	*1952 (95.7)*	2039 (100)	
	Male	48 (7.6)	*586 (92.4)*	634 (100)	
	Total	135 (5.1)	2538 (94.9)	2673 (100)	
**Level of education**					<.001^c^
	Incomplete elementary school	0 (0)	*6 (100)*	6 (100)	
	Complete primary education	3 (17.6)	*14 (82.4)*	17 (100)	
	Incomplete high school	7 (22.6)	*24 (77.4)*	31 (100)	
	Complete high school	32 (11)	*260 (89)*	292 (100)	
	Higher education (or studying)	51 (4.5)	*1095 (95.5)*	1146 (100)	
	Postgraduate studies	42 (3.6)	*1139 (96.4)*	1181 (100)	
	Total	135 (5.1)	2538 (94.9)	2673 (100)	
**Are you a health professional?**					.001^b^
	No	101 (6.1)	*1551 (93.9)*	1652 (100)	
	Yes	34 (3.3)	*987 (96.7)*	1021 (100)	
	Total	135 (5.1)	2538 (94.9)	2673 (100)	
**Have you experienced symptoms of COVID-19?**					.99
	No	114 (5.1)	*2142 (94.9)*	2256 (100)	
	Yes	21 (5)	*396 (95)*	417 (100)	
	Total	135 (5.1)	2538 (94.9)	2673 (100)	
**Did you have a confirmed diagnosis for COVID-19?**					.77
	No	131 (5)	*2473 (95)*	2604 (100)	
	Yes	4 (5.8)	*65 (94.2)*	69 (100)	
	Total	135 (5.1)	2538 (94.9)	2673 (100)	

^a^Data in italics were the most frequent.

^b^Found to be statistically significant by the chi-square test.

^c^Found to be statistically significant by the likelihood ratio test.

## Discussion

### Principal Findings

The main aims of this study were to describe the sociodemographic variables, determine the profiles of individuals compliant to the use of face masks, and compare them between younger and older people. The majority of the 2378 participants in the group aged ≤59 years were women (n=1833, 77.1%), were living in the southeast region (n=2060, 86.6%), and had postgraduate degrees (n=1062, 44.7%). In the case of the 295 individuals aged ≥60 years, the majority were women (n=206, 69.8%), were living in the southeast region (n=271, 91.9%), and had completed or were still completing higher education (n=121, 41%). In addition, the majority of participants in both groups have been using face masks; this was significantly influenced by sex, level of education, and whether the participant was a health professional or not.

This study has two major findings. Nearly 30% to 40% of the participants were health professionals, and 80% to 90% were from São Paulo, that is, people who have good health literacy and educational level and those who are living in regions with the highest socioeconomic level in Brazil. Bambra et al [20] reported that social, economic, demographic differences and inequalities, and lack of access to health care have implications in any pandemic recorded in history, including COVID-19. Dowd et al [9] pointed out that understanding the profile of sociodemographic variables are important for all governments to rapidly make policies to mitigate the negative effects of the COVID-19 pandemic. Through sociodemographic studies, it was possible to verify that the most serious cases and deaths were prevalent among older adults and those with comorbidities. This is important information for health care systems worldwide.

Hence, tracing the sociodemographic profile of different populations worldwide to outline short-, medium-, and long-term positive coping strategies (eg, focused on improving physical and mental health) to help mitigate the COVID-19 pandemic will help decrease inequalities in access to social and health services for future generations. It is interesting to note that hospitalizations and mortality are higher in men than in women and that men are more inclined to smoke and thus have the potential for poorer respiratory outcomes in COVID-19 infection [21].

Regarding the use of face masks, in general, the majority of the participants in this study, both the general population and health professionals, reported adherence to this measure during the course of the COVID-19 pandemic. The factors that significantly influenced this relevant control measure were sex, level of education, and whether the participant is a health professional or not. We highlight three clinical trials, but only one of them is directly related to the use of facial masks as a central measure to control the spread of COVID-19 infection. Bundgaard et al [22] conducted a nonblind, randomized, controlled trial to investigate whether the use of facial masks could reduce the risk of SARS-CoV-2 infection. They included adults (aged >18 years, n=6000) without previously confirmed COVID-19 or symptoms suggestive of COVID-19 who spent more than 3 hours a day outside the home with exposure to other people. The authors concluded that the use of a face mask could be a protective factor for the user against SARS-CoV-2 infection. However, more evidence is needed through robust clinical trials to provide consistent scientific evidence for recommendations from health authorities worldwide and in the future. Liang et al [23] through a meta-analysis concluded that the use of masks is an auxiliary method and measure of health and prevention in relation to the outbreak of COVID-19 and highlighted that these protective health measures are impacted by the level of knowledge and health literacy, [24] and different beliefs, moral values, and even conditions of access to health.

### Final Considerations

In this study, women (*P*<.001), health workers (*P*=.001), and people with a higher level of education (*P*<.001) had greater adherence in relation to the use of masks. A potential correlation was found between gender, where there was a higher incidence of disease in men, and there has been a globally observed shorter life expectancy in men as compared to women [25].

Determining sociodemographic profiles and identifying the factors that favored the use of face masks, especially in São Paulo, does not reflect the actual scenario in Brazil, which is a country with many social inequalities, but provides subsidies to study other regions from the same point of view, which can assist in facing the current scenario and future health crises.

### Study Limitations and Practical Applications

This study has limitations. Using social media to collect the data may have influenced the study sample, as many participants are health workers and living in the state of São Paulo, the most economically developed region in Brazil. Nevertheless, we were able to gather an expressive sample. In view of the results of our study, we believe that the implementation of health care policies aimed at certain age groups, such as older adults, and populations, for example, men and people who are not health professionals, can increase the compliance to disease prevention measures, for example, the use of face masks, since these populations are heterogeneous from the health care point of view.

Other limitations were the absence of some items in our questionnaire regarding family size (number of people living in the same house), the type of mask used (which can be difficult information to access for those people who are not health professionals), evaluation of the proportion of infected men and women, and a larger sample of people 60 years or older. One of the difficulties of our study was to obtain a larger sample of older participants. Our expectation was to have a more equalized sample size between younger and older groups, which was not possible. It is necessary to understand why older adults tend to answer questionnaires less than younger people despite the massive dissemination of the instrument to this audience. Finally, we did not include questions regarding the smoking habits and alcohol consumption (and other health habits) of younger and older people. These are factors that can impact the general health status and outcome of a COVID-19 infection in cases of abuse and excessive or constant use. Despite this, these are areas of study that need to be further explored and may be the focus of future studies.

Finally, education actions, carried out by health workers, for health promotion and disease prevention can be stimulated and carried out increasing the health literacy of the population, which will provide individualized and effective actions.
